# Establishment and Application of a High Throughput Screening System Targeting the Interaction between HCV Internal Ribosome Entry Site and Human Eukaryotic Translation Initiation Factor 3

**DOI:** 10.3389/fmicb.2017.00977

**Published:** 2017-05-29

**Authors:** Yuying Zhu, Pei Huang, Na Yang, Rui Liu, Xueting Liu, Huanqin Dai, Lixin Zhang, Fuhang Song, Chaomin Sun

**Affiliations:** ^1^Key Laboratory of Experimental Marine Biology, Institute of Oceanology, Chinese Academy of SciencesQingdao, China; ^2^Laboratory for Marine Biology and Biotechnology, Qingdao National Laboratory for Marine Science and TechnologyQingdao, China; ^3^College of Earth Science, University of Chinese Academy of SciencesBeijing, China; ^4^Key Laboratory of Pathogenic Microbiology and Immunology, Institute of Microbiology, Chinese Academy of SciencesBeijing, China; ^5^State Key Laboratory of Bioreactor Engineering, East China University of Science and TechnologyShanghai, China

**Keywords:** hepatitis C virus, IRES, eIF3, high throughput, enhancer, inhibitor

## Abstract

Viruses are intracellular obligate parasites and the host cellular machinery is usually recruited for their replication. Human eukaryotic translation initiation factor 3 (eIF3) could be directly recruited by the hepatitis C virus (HCV) internal ribosome entry site (IRES) to promote the translation of viral proteins. In this study, we establish a fluorescence polarization (FP) based high throughput screening (HTS) system targeting the interaction between HCV IRES and eIF3. By screening a total of 894 compounds with this HTS system, two compounds (Mucl39526 and NP39) are found to disturb the interaction between HCV IRES and eIF3. And these two compounds are further demonstrated to inhibit the HCV IRES-dependent translation *in vitro*. Thus, this HTS system is functional to screen the potential HCV replication inhibitors targeting human eIF3, which is helpful to overcome the problem of viral resistance. Surprisingly, one compound HP-3, a kind of oxytocin antagonist, is discovered to significantly enhance the interaction between HCV IRES and eIF3 by this HTS system. HP-3 is demonstrated to directly interact with HCV IRES and promote the HCV IRES-dependent translation both *in vitro* and *in vivo*, which strongly suggests that HP-3 has potentials to promote HCV replication. Therefore, this HTS system is also useful to screen the potential HCV replication enhancers, which is meaningful for understanding the viral replication and screening novel antiviral drugs. To our knowledge, this is the first HTS system targeting the interaction between eIF3 and HCV IRES, which could be applied to screen both potential HCV replication inhibitors and enhancers.

## Introduction

Hepatitis C virus (HCV) infection is a major cause of clinical burden worldwide because many patients can develop complications including chronic hepatitis, liver cirrhosis, and hepatocellular carcinoma (Sun et al., 2010). The genome of HCV is a single positive-strand RNA and consists of a single open reading frame flanked by 5′-untranslated region (UTR) and 3′-UTR. Notably, the internal ribosome entry site (IRES) of HCV located in the genomic 5′-UTR determines the formation of the eukaryotic translation initiation complex at the correct start codon by a cap-independent mechanism (Sun et al., 2013). The translation of the viral genomic RNA is driven by HCV IRES, which can help the virus to evade the host immune response by suppressing canonical cap-dependent translation (Fraser and Doudna, 2007). Thus, the unique function of HCV IRES makes it crucial in the life-cycle of HCV.

Hepatitis C virus is the major etiological agent of non-A and non-B hepatitis without any available vaccine, and has infected 3% of the World’s population, of whom approximately 500,000 people die each year (Poynard et al., 2003; Lozano et al., 2013). In the previous time, the most widely used anti-HCV therapy was a combination of pegylated interferon alpha and ribavirin with low success rate and high side effects (Moradpour et al., 2007). Recently, several effective direct-acting antivirals have been developed, including NS3/4A protease inhibitors GS-9256 and GS-9451, NS5A protein inhibitor ledipasvir, NS5B (nucleoside-type) polymerase inhibitor sofosbuvir, and NS5B (non-nucleoside-type) polymerase inhibitor tegobuvir (De Clercq, 2014). Sustained virologic response for HCV infection of these drugs have been observed, and drug combinations of two or more DAAs belonging to any of the four categories are developing (De Clercq and Li, 2016). Sofosbuvir is proposed to be one most effective anti-HCV drug, however, resistant variants to sofosbuvir were found at different frequencies in worldwide HCV-1b sequences, and the high frequency of double mutation L159F-C316N observed in Brazilian HCV-1b patients (Peres-da-Silva et al., 2017). Therefore, the resistance to the present effective anti-HCV drugs is still a potential problem for the future anti-viral therapy. Alternatively, targeting a host factor rather than a viral protein might avoid the drug resistance for the virus (Sun et al., 2010). Thus, development of new classes of antiviral compounds targeting host factors is always needed.

Viruses are intracellular obligate parasites and the host cellular machinery is usually recruited for their replication (Ndjomou et al., 2009). Understanding the host–pathogen interactions is essential for the development of efficient therapies against viruses (Angulo et al., 2016). Notably, HCV has been shown to manipulate translation initiation to further their replication, and human translation initiation factor eIF3 is one of the essential factors (Sun et al., 2013). Human eIF3, an 800 kDa molecular mass assembly of 13 proteins (eIF3a–eIF3m), plays an important role in translation initiation mediated by the HCV IRES (Sun et al., 2011). Human eIF3 and the 40S ribosomal subunit interact specifically with the defined secondary and tertiary structural elements of the HCV IRES (Fraser and Doudna, 2007). These structural interactions drive the genomic RNA for translation initiation at the correct start codon without the need for assembling the translation initiation machinery and mRNA scanning (Sun et al., 2011). With the success of reconstitution of human eIF3 in *E. coli* (Sun et al., 2011), the interaction between eIF3 and HCV IRES was well elucidated (Sun et al., 2013). Sun et al. (2013) identified a surprising interaction between an HLH motif in eIF3a and the IIIb stem-loop of the HCV IRES. Moreover, this interaction is a key issue for the formation of HCV IRES-dependent preinitiation complex and the assembly of functional 80S ribosome (Sun et al., 2013). The local nature of this contact has potentials to serve as a new target for developing effective drugs against HCV IRES-mediated translation initiation (Sun et al., 2013).

In this study, an fluorescence polarization (FP) based high throughput screening (HTS) system targeting the interaction between HCV IRES and eIF3 is established to screen the potential inhibitors of HCV replication. Two compounds Mucl39526 and NP39 are found to interrupt the interaction between HCV IRES and eIF3 by this HTS system, and these two compounds have potentials to develop the antiviral drugs. On the other hand, one compound HP-3, a kind of oxytocin antagonist, is discovered to significantly promote the interaction between eIF3 and HCV IRES. HP-3 is further demonstrated to enhance the HCV IRES-dependent translation both *in vitro* and *in vivo*, which strongly suggests that HP-3 has potentials to promote HCV replication and it could be developed to some kind of HCV replication enhancer. To our knowledge, this is the first HTS system targeting the interaction between eIF3 and HCV IRES, which could be applied to screen both potential HCV replication enhancers and inhibitors in the future.

## Materials and Methods

### Expression and Purification of eIF3 Octamer (a^∗^c^∗^fhelmk)

For the expression of eIF3 octamer (a^∗^c^∗^fhelmk), plasmids 2D containing His_6_-GST-e/His_6_-GST-l/His_6_-MBP-m/His_6_-GST-k and 2E containing His_6_-SUMO-c^∗^/a^∗^/His_6_-MBP-h/His_6_-GST-f were co-transformed into *E. coli* Rosetta (DE3) competent cells (EMD Biosciences), with 100 mg/L ampicillin and 50 mg/L kanamycin for selection. The octamer was purified as described previously (Sun et al., 2011). Fractions of pure protein was pooled together and concentrated by ultrafiltration using Vivaspin^®^ Turbo 15 (MWCO 100,000 Dalton, Sartorius) before stored at -80°C.

### Transcription and End Labeling of HCV IRES

The HCV IRES used in this study includes nucleotides 39–352 of the HCV subtype 1b (con1) genomic RNA. Plasmid pUC19IRES containing sequence of HCV IRES was used for *in vitro* transcription (Sun et al., 2011). The plasmid was linearized by *Bam*HI and used as template for *in vitro* transcription, which was executed with MEGAscript T7 Kit (Thermo Fisher Scientific). Afterward, the acquired RNA was 3′ end labeled with fluorescein-5-thiosemicarbazide (SIGMA-ALDRICH, CAS number 76863-28-0) as described previously (Kieft et al., 2001; Sun et al., 2013). Briefly, the labeling of HCV IRES was successively processed by RNA oxidation, KCl precipitation, and dye labeling and cleaning up. RNA oxidation reaction contained 240 μg HCV IRES, 95 μL 0.42 M NaIO_4_, 13.3 μL 3 M NaOAc (pH 5.2) in 400 μL total volume, and the reaction was performed in dark for 90 min at room temperature with shaking. Then the reaction was stopped by adding 11 μL 4M KCl and incubated on ice for 10 min. The precipitate was removed by centrifugation at 12,000 rpm for 1 min, and salt in the supernatant was removed using Sephadex G-25 column (GE Healthcare). The oxidized RNA was labeled with 5–100 mM Fluorescein-5-thiosemicarbazide in a buffer of 0.1 M NaOAc (pH 5.2) in dark for 4 h at room temperature with shaking. Finally, the excess fluorophore was removed by phenol extraction for two times, and the labeled RNA was purified by G-25 column and ethanol precipitation. The obtained labeled RNA was stored at -80°C. The labeled HCV IRES was annealed at 65°C for 5 min followed by cooling to room temperature for at least 10 min in THEMK buffer (34 mM Tris, 66 mM HEPES, 0.1mM EDTA, 2.5 mM MgCl_2_, 75 mM KCl) (Sun et al., 2011).

### Establishment of the FP Based HTS System

Fluorescence polarization assays were carried out using 96-well black plates on a Tecan M1000Pro Multiscan Spectrum, with detection wavelength of excitation 492 nm and emission 516 nm, and the final volume of each reaction was 50 μL. For each measurement plate, three blank wells containing THEMK buffer and three reference blank wells containing 20 nM fluorescent HCV IRES in THEMK buffer were included. Firstly, to select the optimal protein concentration, different concentrations of eIF3 octamer were added to the assay system containing 20 nM fluorescent HCV IRES in THEMK buffer. Then the impacts of reaction time and DMSO concentration were detected. The detection range of reaction time and DMSO concentration is 0–12 min and 0–1.6%, respectively. Non-fluorescence labeled HCV IRES (400 nM) was used as positive control to competitively combine with eIF3 octamer. Z factor was used to evaluate the quality of the FP system (Z′ = 1 – (3 SD of positive control + 3 SD of negative control)/| mean of positive control - mean of negative control|) (Sui and Wu, 2007), and 40 repeats of positive control and 40 repeats of negative control were tested for Z factor calculating.

For the optimized FP system, 1000 compounds/crude extracts were screened and the screening concentration was set as 100 μM (compounds) or 40 μg/mL (crude extracts) with three repeats for each treatment. For positive hits from the primary screening, dose-dependent assays were carried out with FP method. Dose-dependent curve was generated by GraphPad Prism 5.01 software.

### Electrophoretic Mobility Shift Assay (EMSA)

Electrophoretic mobility shift assay was used to identify binding activity of HCV IRES and eIF3 octamer, or the effects of active agents to HCV IRES and eIF3 octamer interaction (Sun et al., 2011). The reaction system contained 20 nM labeled HCV IRES, different concentration of eIF3 octamer in the presence or absence of active agent. The reaction was carried out at 25°C for 10 min, and then resolved by 1% native agarose gel at 4°C, 40 V for 40 min in the THEMK buffer. Resulting gels were visualized by fluorescence imaging using Typhoon FLA 9500 with the wavelength of 492 nm.

### *In Vitro* Translation

The *in vitro* translation assays were conducted with Rabbit Reticulocyte Lysate System (Promega) as previously described (Zhou et al., 2013). A dual luciferase plasmid pHCV-dual containing a cap-dependent *Renilla* luciferase gene and an HCV IRES-dependent Firefly luciferase gene was used for the assays. The plasmid was first linearized with *Xba*I and purified as described above. Then the mMESSAGE mMACHINE^®^ T7 Kit (Thermo Fisher Scientific) was used to produce capped RNA with linearized plasmid as template, and the RNAs were subsequently tailed with Poly (A) Tailing Kit (Thermo Fisher Scientific). For the *in vitro* translation assays, 1 μg RNA was used for each 50 μL reaction, and the translation efficiencies were determined by detecting signals of *Renilla* luciferase and Firefly luciferase with Dual-Luciferase^®^ Reporter Assay System (Promega). The final concentration of Mucl39526 and NP39 used for *in vitro* translation assay is 40 μg/mL. The final concentration of HP-3 used for *in vitro* translation assay is 100 μM.

### *In Vivo* Translation Assay in Huh 7.5 Cell Line

Human hepatoma cell line, Huh 7.5, was maintained in Dulbecco’s modified Eagle’s medium (DMEM) supplemented with 100 U/mL penicillin, 100 μg/mL streptomycin, non-essential amino acids, and 10% fetal bovine serum (FBS) at 37°C in 5% CO_2_ (Tsukimoto et al., 2015). For cell viability assay, the cells were seeded in 96-well tissue culture plate and incubated for 12 h, then treated with different concentrations of HP-3. After 24 h incubation, MTT solution (0.5 mg/mL) was added to the wells, incubated for 4–5 h at 37°C, and formazan crystals in viable cells were dissolved in 100 μL DMSO. The soluble formazan was spectrophotometrically quantified with a Tecan M1000Pro Multiscan Spectrum at 490 nm. Dose-dependent curve was generated by GraphPad Prism 5.01 software.

For cell-based bicistronic reporter assay, Huh 7.5 cells were seeded in 24-well tissue culture plate and incubated to 70–90% confluence, and then the dual luciferase plasmid pHCV-dual was transfected with TransIn EL Transfection Reagent (TransGen Biotech) in accordance with the manufacturer’s instructions. Different concentration of HP-3 was added to the system after 5 h of transfection, and incubated for 36 h before the luciferase activities were detected. Plasmid pcDNA3.1 was added to the transfections in order to achieve the same total amount of plasmid DNA per transfection. The luciferase activity was evaluated using Dual Luciferase Reporter Assay System (Promega) on a Tecan M1000Pro Multiscan Spectrum.

### SHAPE Modification Chemistry

The full length HCV IRES was amplified from plasmid pUC19IRES with forward primer 5′-TAATACGACTCACTATAGGCCTTCGGGCCAACTCCCCTGTG AGGAACTACTG-3′ and reverse primer 5′-GAACCGGACCGAAGCCCGATTTGGTTTTTCTTTGAGGTTCAGGATTTGTGCTC-3′. The PCR product was purified and used as template for *in vitro* transcription with MEGAScript T7 Kit (Thermo). For SHAPE analysis, the HCV IRES (25 pmol for each reaction) was heated at 95°C for 3 min, then placed on ice for 1 min, and followed by refolding in THEMK buffer with final volume of 44 μL at 37°C for 10 min. The folded RNA was incubated with 1 μL HP-3 (10 mM) at room temperature for 10 min. Subsequently, 5 μL of 1M7 (65 mM) dissolved in DMSO or DMSO only was added to each reaction and incubated at 37°C for 70 s (Sun et al., 2013). The reactions were extracted two times with equal volume of phenol/chloroform and once with equal volume of chloroform. Then two volumes of ethanol was added to precipitate RNAs at -80°C overnight and resuspended in 12 μL nuclease-free water. For primer extension, 1 μL of 10 μM fluorescently labeled DNA primer (5′-FAM-labeled GAACCGGACCGAAGCCCG-3′) was added to 12 μL of modified RNA; for RNA sequencing reaction, equal amount of labeled primer was added to 11 μL unmodified HCV IRES (10 pmol). The primer was annealed to the RNA by heating at 65°C for 5 min and incubated on ice for 1 min. The 6 μL of SuperScript enzyme mix (including 4 μL 5 × SuperScript buffer, 1 μL 0.1 mM DTT, 0.1 μL 10 mM dATP, 0.1 μL 10 mM dTTP, 0.1 μL 10 mM dCTP, 0.1 μL 10 mM dITP, and 0.6 μL Nuclease-free water) and 1 μL SuperScript III enzyme were added to the reactions. For sequencing reaction, 1 μL 1 mM ddGTP was also added and incubated at 45°C for 1 min, 52°C for 25 min and 65°C for 5 min. The 4 μL EDTA (50 mM, pH 8.0) was added to quench the reaction, and the obtained cDNA was precipitated by ethanol and washed with 70% ethanol twice (Mortimer and Weeks, 2009). The pellet was dried and resuspended in 10 μL deionized formamide for capillary electrophoresis analysis, meanwhile, an internal size standard LIZ 500 was added. And for each SHAPE analysis, three repeats were executed.

Data were processed as previously described with minor modification (Pang et al., 2011). The FSA files obtained from capillary electrophoresis were first analyzed using PeakScanner v1.0 with parameters of smoothing = none, window size = 25, size calling = local southern, baseline window = 51, peak threshold = 15. Then FAST v1.0 module in RNAstructure v5.2 was used to generate the ladder position file and SHAPE files. The RNA structures were analyzed in RNAstructure web servers with SHAPE intercept parameter of 2.6 kcal/mol and SHAPE slope parameter of -0.8 kcal/mol. Finally, the structures were drawn with RNAViz2 (De Rijk et al., 2003).

### Statistical Analysis

All analysis was performed using Excel 2007 software. Data are presented as mean ± standard deviation (SD). Statistical difference was determined by a paired two-tailed Student’s *t*-test. *P* < 0.05 is considered statistically significant.

## Results

### Establishment of the HTS System Targeting the Interaction between HCV IRES and eIF3 Octamer

**FIGURE 1:**
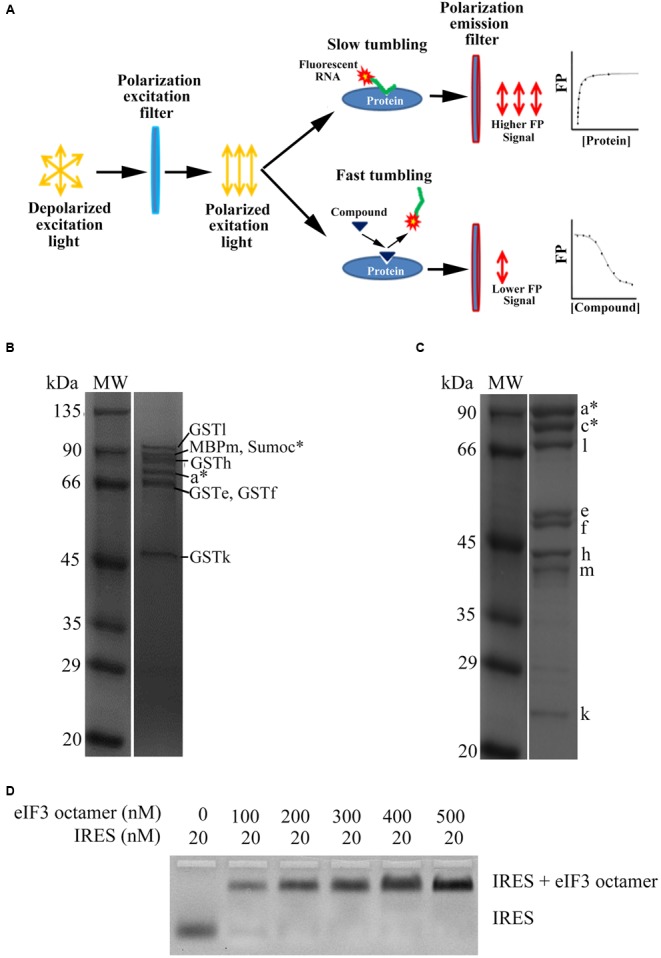
Purification of human eIF3 octamer and binding assays of eIF3 octamer with HCV IRES. **(A)** The schematic diagram of fluorescence polarization based on the interaction between protein and fluorescent labeled RNA. **(B)** Coomassie stained SDS-PAGE gel analysis of purified eIF3 octamer with affinity tags. Molecular weight (MW) markers are shown in kilodaltons. **(C)** Coomassie stained SDS-PAGE gel analysis of cleaved eIF3 octamer. MW markers are shown in kilodaltons. **(D)** Native agarose gel showing binding of the eIF3 octamer to the HCV IRES. The nanomolar concentrations of the octamer and HCV IRES are listed. The reactions were carried out in the presence of 2 μM tRNA to prevent non-specific binding.

The initial purpose of this study is to establish an effective HTS system and screen the potential HCV replication inhibitors targeting the interaction between HCV IRES and eIF3. In our previous study, we succeeded to functionally reconstitute the 13-subunits eIF3 in *E. coli*, and demonstrated that the eIF3 PCI/MPN octamer exhibited similar binding capacities to the HCV IRES (Sun et al., 2011). Therefore, in this study we sought to establish an HTS system based on the *E. coli* reconstituted eIF3 octamer and fluorescein-labeled HCV IRES. Correspondingly, FP technique was used for the HTS system establishment. FP is one of the most widely used techniques in high throughput format assays of clinical chemistry, and its effectiveness has been illustrated by numerous applications in the therapeutic drug monitoring (Zhu et al., 2012). The basic principle of this method is that the fluorophore’s emission can be polarized when excited by plane polarized light, and the polarization level of the fluorophore depends on its molecular size. Small molecules are prone to fast tumbling due to Brownian motions, thus the achieved polarization emits easily and produces a low FP signal. However, when the small molecule binds to large molecule, a relative slower tumbling would be resulted and a higher FP signal could be correspondingly detected (**Figure 1A**) (Ansideri et al., 2016). Therefore, in this study, HCV IRES was labeled with fluorophore, and a higher FP signal would be obtained once it binds to eIF3. Consistently, the FP value will markedly drop or increase while the interaction between HCV IRES and eIF3 is disrupted or enhanced by some compound, respectively.

The eIF3 octamer used in this study was co-expressed by PCI/MPN subunits (subunits eIF3a^∗^c^∗^efhklm, asterisks indicating the truncations), which has a molecular mass of approximately 400 kDa. Here, subunits eIF3a and eIF3c truncated to remove likely flexible regions of the proteins as described previously (Sun et al., 2011). During the purification of the octamer, tagged protein was first purified with GST column, and six bands were obtained in the SDS-PAGE gel, which included GSTl, MBPm/SUMOc^∗^, MBPh, a^∗^, GSTe/GST3f and GST3k (**Figure 1B**). The affinity tags were cleaved by TEV protease and thereafter ran through anion exchange and gel filtration columns to get the pure eIF3 octamer (**Figure 1C**). The HCV IRES was transcribed and labeled at 3′-terminal with Fluorescein-5-thiosemicarbazide as described in the “Materials and Methods” section. The binding capacity of labeled HCV IRES with purified eIF3 octamer was verified by EMSA assay. The results showed that the biochemically stable PCI/MPN octamer bound tightly to the HCV IRES and gave an apparent shift compared to the free RNA (**Figure 1D**), which is consistent with the results described previously (Sun et al., 2011). Taken together, our results indicated that the reconstituted eIF3 octamer and fluorescein-labeled HCV IRES are functional for FP assays.

**FIGURE 2:**
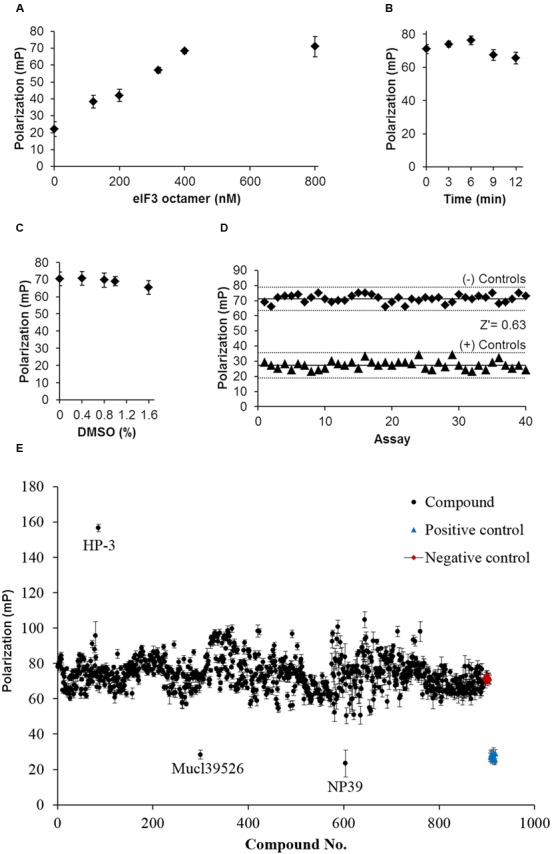
Establishment and optimization of FP-based HTS system targeting the interaction between HCV IRES and eIF3 octamer. **(A)** Optimization of eIF3 octamer concentration in the presence of 20 nM labeled HCV IRES for HTS. **(B)** Optimization of reaction time for HTS system. **(C)** Optimization of DMSO concentrations for HTS system. **(D)** Fluorescence polarization of 40 positive control assays [non-labeled HCV IRES (+)) and 40 negative control assays (DMSO only (–)]. Solid lines represented means and dotted lines three times the standard deviations of the mean of all assays. **(E)** Screening results from the FP-based HTS system targeting the interaction between HCV IRES and eIF3 octamer.

To optimize the concentration of eIF3 octamer used in FP system, 20 nM labeled HCV IRES and different concentrations of eIF3 octamer were added to each reaction and performed FP assay. As shown in **Figure 2A**, the FP value reached a plateau when 400 nM of eIF3 octamer was added in the reaction. Therefore, 400 nM was determined as the concentration of eIF3 octamer used for the following screening. Another two important factors for HTS system, reaction time and DMSO concentration, were also optimized. Reaction time from 0 to 12 min and DMSO concentration from 0 to 1.6% (v/v) were checked, respectively. The results showed that there were no significant difference of FP values in the reaction time and DMSO concentration ranges mentioned above (**Figures 2B,C**). After conditions optimization, FP-based binding assays were then performed in a high throughput format to judge necessary precision and reproducibility. To judge well-to-well variation by FP-based binding assays, 40 negative controls [1% (v/v) of DMSO only] and 40 positive controls (400 nM non-labeled HCV IRES) were performed, which resulted in a Z′ factor of 0.63 (>0.5) (**Figure 2D**) and is considered acceptable for HTS system.

### Active Agents Impacting the Interaction between HCV IRES and eIF3 Octamer Discovered by the HTS System

A set of 1000 compounds including clinical drugs or natural products were tested by the established FP system in a 96-well plate format. Firstly, the compounds with autofluorescence at the similar wavelengths of fluorophore (absorbing at wavelengths of excitation 492 nm and emission 516 nm) employed for the FP system were excluded because of the strong background as described previously (Sui and Wu, 2007). For the remained active agents, three parallel repeats were performed for the primary FP screening. Out of the 894 samples screened, three active agents (HP-3, Mucl39526, and NP39) exhibited FP signals significantly different from other library samples (**Figure 2E**). As for these three active agents, two crude extracts derived from marine actinobacteria, Mucl39526 and NP39, could decrease the FP signals. Thus, they were proposed to disrupt the interaction between HCV IRES and eIF3 octamer. In contrast, one pure compound HP-3 dramatically increased the FP signals, and it was proposed to promote the interaction between HCV IRES and eIF3 octamer. To disclose the potential functions of these three active agents for HCV replication, they were selected for further studies.

### Active Agents Mucl39526 and NP39 Disrupt the Interaction between HCV IRES and eIF3 Octamer

**FIGURE 3:**
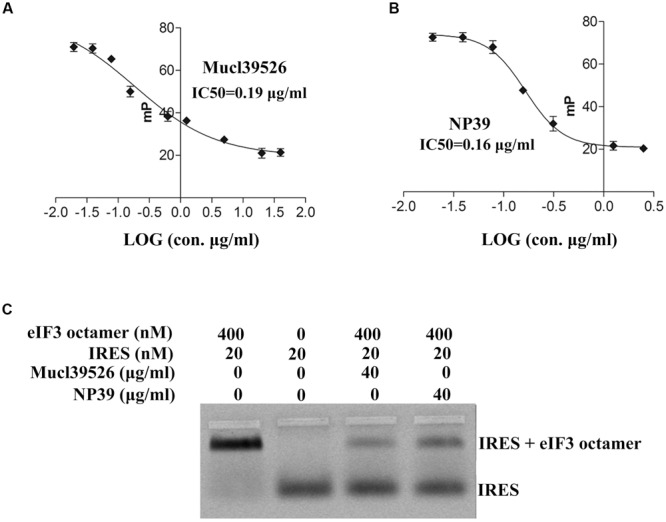
Active agents Mucl39526 and NP39 disrupt the interaction between HCV IRES and eIF3 octamer. **(A)** IC50 assays of Mucl39526 toward the interaction between HCV IRES and eIF3 octamer. **(B)** IC50 assays of NP39 toward the interaction between HCV IRES and eIF3 octamer. **(C)** Native agarose gel showing disrupting of the interaction between HCV IRES and eIF3 octamer and HCV IRES by Mucl39526 and NP39. The nanomolar concentrations of the eIF3 octamer, HCV IRES, Mucl39526, and NP39 are listed. The reactions were carried out in the presence of 2 μM tRNA to prevent non-specific binding.

The inhibition of Mucl39526 and NP39 toward the interaction between HCV IRES and eIF3 was further evaluated by IC_50_. And the IC_50_ of Mucl39526 and NP39 was determined to be 0.19 and 0.16 μg/mL by FP assay, respectively (**Figures 3A,B**). To further validate the effects of Mucl39526 and NP39 on the interaction of HCV IRES and eIF3 octamer, EMSA assay was performed. The reaction system included 20 nM HCV IRES, 40 μg/mL Mucl39526 or NP39, and 400 nM eIF3 octamer. Notably, in the presence of Mucl39526 or NP39, the bands for HCV IRES–eIF3 octamer complex were weakened, while the bands for free IRES RNA became obvious (**Figure 3C**). These results strongly indicated that Mucl39526 and NP39 could interrupt the interaction between HCV IRES and eIF3 octamer.

**FIGURE 4:**
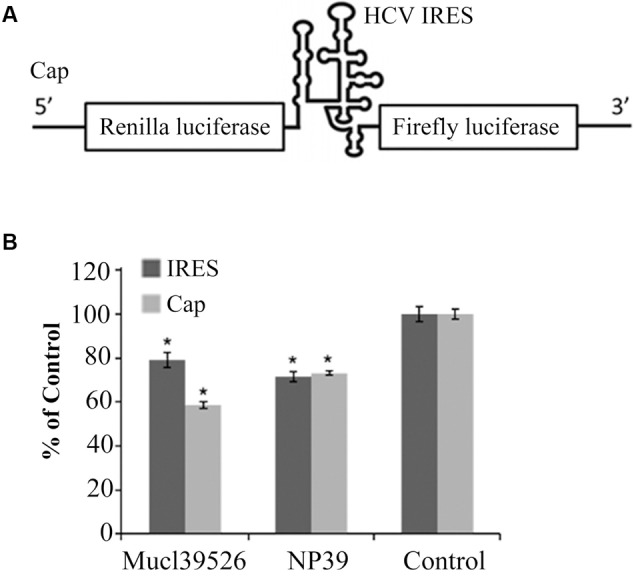
Effects of Mucl39526 and NP39 on HCV IRES-dependent or cap-dependent translation *in vitro*. **(A)** Bicistronic reporter used for compound testing in a coupled *in vitro* transcription-translation assay. **(B)** Effects of Mucl39526 and NP39 (40 μg/mL) on HCV IRES-dependent or cap-dependent translation. ^∗^*P* < 0.05 compared with control.

The presence of Mucl39526 or NP39 inhibits the recruitment of eIF3 by HCV IRES, therefore, we next explored the effects of Mucl39526 or NP39 on the HCV IRES-dependent translation via *in vitro* translation system. The *in vitro* translation assay was conducted with a bicistronic reporter that carries a Firefly luciferase reporter driven by a functional HCV IRES and a cap-dependent *Renilla* luciferase reporter (**Figure 4A**). As the results shown in **Figure 4B**, Mucl39526 and NP39 could significantly inhibit HCV IRES-dependent translation (signals of Firefly luciferase), which is consistent with the results of FP assays and EMSA analysis. However, Mucl39526 and NP39 could also inhibit the cap-dependent translation (signals of *Renilla* luciferase), which indicates that the translation inhibition of these two active agents is not HCV IRES-specific. It is known that cellular mRNAs are predominantly translated in a cap-dependent manner, however, a subset of cellular mRNAs initiate their translation in an IRES-independent manner (Kolekar et al., 2016). Thus, Mucl39526 and NP39 could also inhibit the translation of some cellular mRNAs with an IRES-independent manner. Nonetheless, our results demonstrate that the HTS system targeting the interaction between HCV IRES and eIF3 is functional to screen the potential HCV replication inhibitors. And we could screen more compounds with this HTS system to obtain the potential antiviral drugs in the future.

### HP-3 Promotes the Interaction between HCV IRES and eIF3

**FIGURE 5:**
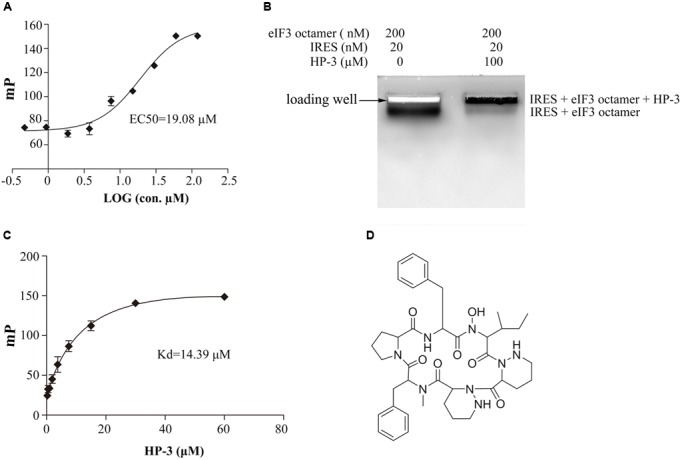
HP-3 promotes the interaction between HCV IRES and eIF3 octamer. **(A)** Concentration response of compound HP-3 on the fluorescence polarization of HCV IRES-eIF3 complex. Data (*n* = 3) were fitted to a log (against) vs. response equation (variable slope). **(B)** Native agarose gel showing binding of the HP-3 to the complex of eIF3 octamer and HCV IRES. The nanomolar concentrations of the eIF3 octamer, HCV IRES and HP-3 are listed. The reactions were carried out in the presence of 2 μM tRNA to prevent non-specific binding. **(C)** *K*_d_ analysis of HP-3 and HCV IRES using FP assay. **(D)** Chemical structure of HP-3.

Notably, the compound HP-3 markedly increased the FP value, thus, it is proposed to enhance the HCV IRES–eIF3 interaction. Taken into account the possibility of HP-3 to promote HCV IRES-dependent translation, it was selected for further study to explore the potential functions in HCV replication. The promotion of HP-3 toward the interaction between HCV IRES and eIF3 was further evaluated by EC_50_, and the EC_50_ of HP-3 was determined to be 19.08 μM by FP assay (**Figure 5A**). To further validate the effect of HP-3 on the interaction of HCV IRES and eIF3 octamer, EMSA assay was performed. The reaction system included 20 nM HCV IRES, 100 μM HP-3, and 200 nM eIF3. Notably, the band for free RNA which disassociated from the complex during electrophoresis was almost completely absent in the presence of HP-3, which strongly indicated that HP-3 could stabilize the complex of HCV IRES–eIF3 octamer (**Figure 5B**). Importantly, the complex of HCV IRES–eIF3 octamer together with HP-3 ran much slower than that of HCV IRES–eIF3 octamer, which suggested that the presence of HP-3 might change the conformation of the complex and this complex almost stuck in the well without any obvious shift compared to that in the absent of HP-3 (**Figure 5B**).

Next, we asked whether HP-3 directly interacted with HCV IRES. Thus, the interaction between HP-3 and HCV IRES was further checked by FP assay. Different concentrations of HP-3 was added to 50 μL THEMK buffer containing 20 nM Fluorescein-5-thiosemicarbazide labeled HCV IRES, and the corresponding FP signals were detected as described above. As expected, the presence of HP-3 increased the FP signals in a dose-dependent manner with a K_d_ of 14.39 μM (**Figure 5C**), which indicated that HP-3 directly interacted with HCV IRES.

Based on the information provided by our collaborator, compound HP-3 is derived from *Streptomyces* sp. MS 110128, which belongs to class of cyclic hexapeptide with a molecular formula of C_40_H_54_N_8_O_7_. The chemical ID of HP-3 is L156373 in CHEMnetBASE database and has a structure as illustrated in **Figure 5D**. To our big surprise, studies showed that HP-3 was a kind of oxytocin/arginine vasopressin (OT/AVP) antagonist, with some selectivity *vs.* liver AVP-V1 and kidney AVP-V2 receptors (Pettibone et al., 1989).

### HP-3 Promotes the HCV IRES-Dependent Translation *In Vitro* and *In Vivo*

**FIGURE 6:**
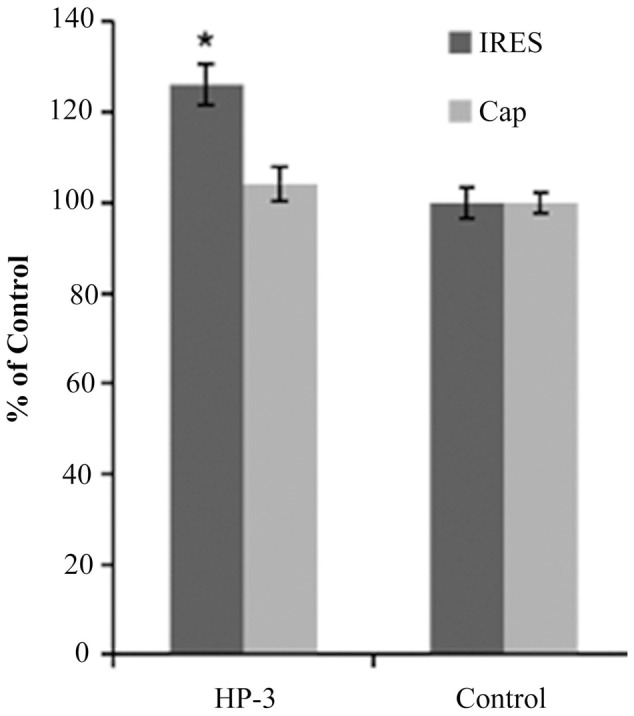
Effects of HP-3 on HCV IRES-dependent or cap-dependent translation *in vitro*. ^∗^*P* < 0.05 compared with control.

The presence of HP-3 promotes the recruitment of eIF3 by HCV IRES, therefore, we next explored the effects of HP-3 on the HCV IRES-dependent translation via *in vitro* translation system as described above. The results showed that HP-3 could selectively enhance HCV IRES-dependent translation (signals of Firefly luciferase) while not affecting cap-dependent translation (signals of *Renilla* luciferase) (**Figure 6**), which is consistent with the conclusion that HP-3 changed the HCV IRES structure to facilitate translation initiation.

**FIGURE 7:**
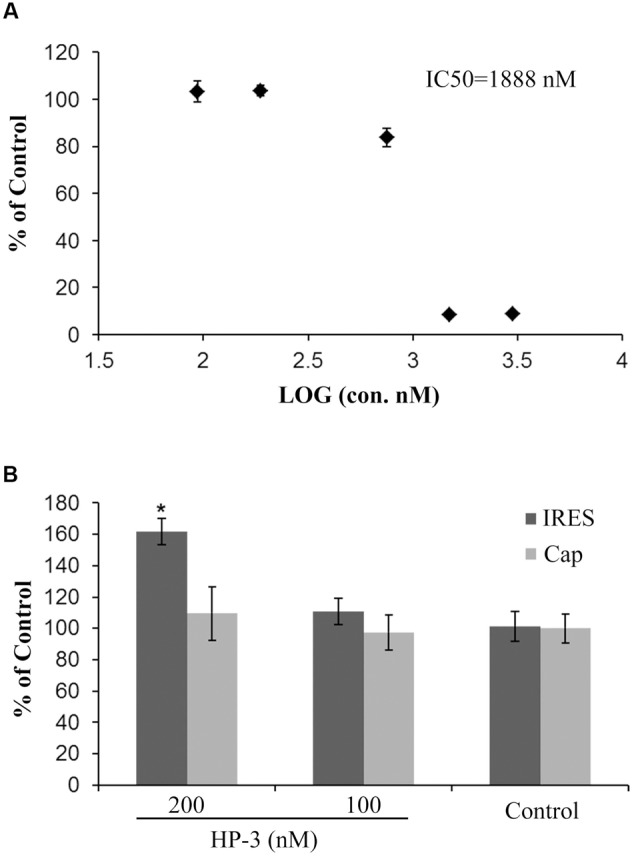
Effects of HP-3 on HCV IRES-dependent or cap-dependent translation *in vivo*. **(A)** Cell toxicity assay of HP-3 using MTT method. **(B)** Effects of HP-3 (200 nM) on HCV IRES-dependent or cap-dependent translation in cells using transient transfection and luciferase reporter assay. ^∗^*P* < 0.05 compared with control.

Many compounds appeared to have strong activity when the luciferase reporter gene assay alone was considered regardless of the cell viability assay. However, most of the potential antiviral compounds were cytotoxic when these results were evaluated in conjunction with the cell viability assay (Kim et al., 2007). For this reason, it is imperative to perform the *in vivo* assay. Therefore, the impact of HP-3 on the HCV IRES-dependent translation in the cell line of Huh 7.5 was further investigated. Firstly, cell viability assay was detected for HP-3 using MTT method. As shown in **Figure 7A**, the IC_50_ of HP-3 was 1833 nM. To check the effects of HP-3 on HCV IRES-dependent translation inside the cells, two concentrations (100 and 200 nM) of HP-3 without any obvious impact on cell viability were selected to conduct transient transfection and luciferase reporter related assays. A significant stimulation of HCV IRES-dependent translation was observed at the concentration of 200 nM of HP-3 when compared with the control, while cap-dependent translation didn’t have obvious change. However, when the concentration of HP-3 was dropped to 100 nM, no significant promotion of HCV IRES-dependent translation was observed (**Figure 7B**). Altogether, HP-3 promotes the HCV IRES-dependent translation both *in vitro* and *in vivo*.

### HP-3 Induces Conformation Change of HCV IRES for Enhancing HCV IRES-Dependent Translation

**FIGURE 8:**
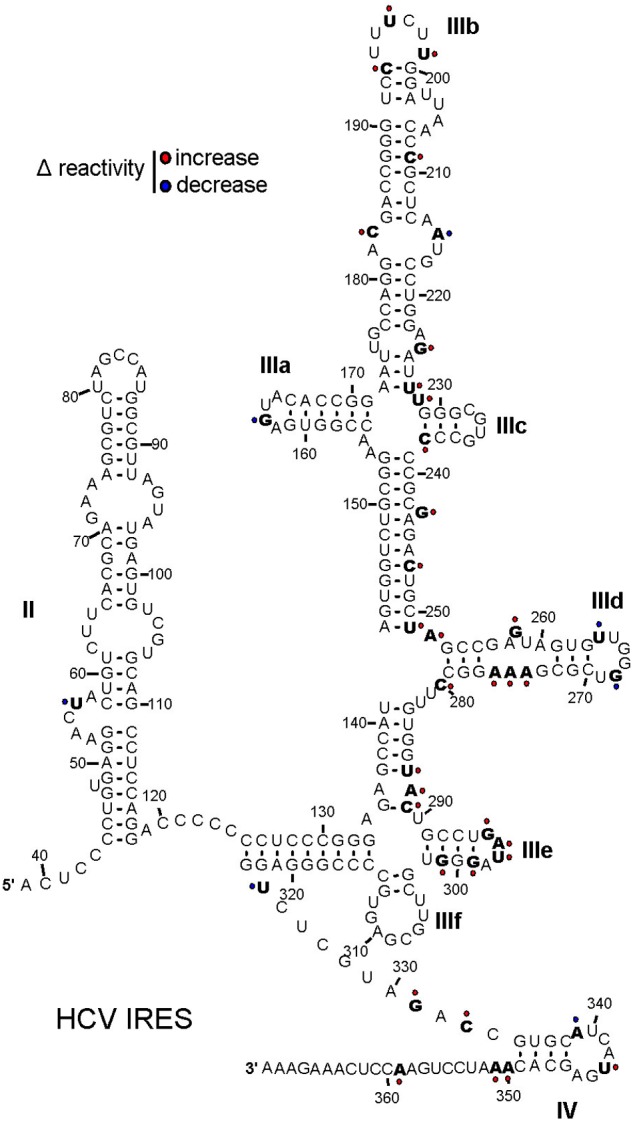
SHAPE-reactivity changes of HCV IRES RNA in the presence of HP-3. SHAPE reactivity differences are mapped onto the secondary structure of the HCV IRES in the presence of compound HP-3, and nucleotides that show increased (red) or decreased (blue) reactivity are indicated.

HP-3 could effectively promote the interaction between eIF3 and HCV IRES and the HCV IRES-dependent translation both *in vitro* and *in vivo*. Furthermore, HP-3 is demonstrated to directly interact with HCV IRES. Next, we sought to check the interaction details between HCV IRES and HP-3, and SHAPE assays were performed to analyze the flexibility of each nucleotide in HCV IRES in the absence and presence of HP-3. SHAPE technology has emerged as one leading method to determine RNA secondary structure at the nucleotide level (Pang et al., 2011). Based on the specificity of the acylation reagent, our results demonstrated a global SHAPE-reactivity increases in the domain IIIabc, domain IIId, and domain IIIe of HCV IRES (**Figure 8**), which indicated that binding of HP-3 could increase the flexibility of these regions. It is noteworthy that the HCV IRES IIIabc region is essential for the interaction of HCV IRES with eIF3 (Sun et al., 2013). The IIId, IIIe domains of HCV IRES are important for the structural rearrangement of the 40S-HCV IRES complex (Angulo et al., 2016). Taken together, HP-3 changes the conformation of HCV IRES and makes it easier recruit translation initiation factors like eIF3 or 40S ribosomal subunit.

## Discussion

Hepatitis C virus was hard to control due to its high prevalence and limited efficacy of interferon-mediated therapies in the previous time (Trahtenherts et al., 2008). In the past few years, several effective inhibitors targeting HCV protease and polymerase were developed (De Clercq and Li, 2016). Sofosbuvir, approved for treatment of HCV-infected patients in combination with ribavirin or with other antivirals, has great activity against all genotypes of HCV and it is a nucleoside analog inhibitor of the HCV NS5B polymerase (Xu et al., 2017). However, resistance to sofosbuvir in genotype 1 and 2 HCV was found and it was conferred by the S282T substitution in NS5B (Xu et al., 2017). Therefore, resistance to the present effective inhibitors is still a potential problem for HCV treatment in the future. Targeting host cellular factors would be one possible solution to solve the problem of resistance mutations in the virus (Sun et al., 2010). Notably, a drug targeting a cellular factor that is essential for the viral life cycle has potentials to inhibit all viruses depending on the same host factor (Kwong et al., 2005; Sun et al., 2010). However, many inhibitors interacting with host cellular factors can’t dissociate their antiviral activity from cytotoxicity. To solve this problem, one possible solution is to disrupt only the interaction between the virus and host cellular factor without affecting the activity of cellular factor toward the host.

In humans, eIF3 plays an important role in HCV IRES-dependent translation initiation (Sun et al., 2013), and factors impacting the interaction between HCV IRES and eIF3 control the viral replication to a great extent. Given that human eIF3 seems to be required for replication of HCV, it appears that eIF3 could be a promising target for anti-HCV drug development (Sun et al., 2013). However, up to now, there is no any available screening system targeting the interaction between HCV IRES and eIF3.

In this study, we successfully established an FP based HTS system targeting the interaction between HCV IRES and eIF3. FP-based method has been reported to be the most precise, reproducible and cost effective for screening of HCV helicase inhibitors where small DNA molecules exist (Mukherjee et al., 2012). And there are instances that applying this technology in analysis of protein-peptide or protein-DNA interactions, however, few reports describe its application in protein-RNA interaction (Tackett et al., 2002; Xu et al., 2003; Rossi and Taylor, 2011). The effectiveness of our FP method has been demonstrated by screening a small pilot of active agents. Together, by combining with EMSA and *in vitro* translation assays (**Figure 3**), the HTS system was proved to be effective in screening inhibitors of HCV IRES-dependent translation in large scale in the future.

To our big surprise, HP-3, one of the compound derived from marine *Streptomyces* sp. MS 110128HP-3 and defined as a kind of OT/AVP antagonist, was found to promote the interaction between HCV IRES and eIF3 octamer based on our HTS results (**Figure 5**). HP-3 showed obvious enhancement to HCV IRES-dependent translation both *in vitro* and *in vivo*, while cellular cap-dependent translation was relatively unaffected by this compound (**Figures 6, 7**), which arouses our interest of its action mechanisms. Surprisingly, HP-3 has binding activity to HCV IRES (**Figure 5**). Moreover, we disclosed the conformation change sites of HCV IRES in the presence of HP-3 by time-resolved RNA SHAPE chemistry. It is known that the functional HCV IRES is composed with three domains (II, III, and IV), where domain III includes IIIa, IIIb, IIIc, IIId, IIIe, and IIIf (Lukavsky, 2009). Importantly, while interacting with eIF3, the entire IRES and domain IIIabc display the same affinity activity (Kieft et al., 2001). Moreover, eIF3a has been demonstrated to make the primary interaction with IRES by binding its IIIb stem loop (Sun et al., 2013). According to the SHAPE results, SHAPE-reactivity was significantly increased in the domain IIIabc of HCV IRES in the presence of HP-3 (**Figure 8**). Therefore, we propose that HP-3 promotes the interaction between eIF3 and HCV IRES by changing the conformation of HCV IRES IIIabc region for eIF3’s easier binding. Additionally, the SHAPE-reactivity was also obviously increased in the domains IIId and IIIe of HCV IRES in the presence of HP-3 (**Figure 8**). Notably, these two domains of HCV IRES are importantly for 40S ribosomal subunit binding (Laletina et al., 2006; Angulo et al., 2016). The loop IIId-ES7S (18S rRNA h26 helix) interaction triggers a structural rearrangement of the 40S/HCV-IRES (Angulo et al., 2016). Thus, it is reasonable to propose that the promotion of HCV IRES-dependent translation might be a result of enhancing recruitments of both eIF3 and 40S ribosomal subunit. Correspondingly, the compound HP-3 might be useful to develop some kind of enhancer for the HCV replicon transfection in the future study.

Hepatitis C virus is a kind of stubborn virus to control. There are still many unknown factors could enhance replication of the virus, which creates more troubles for effective clinical therapy. HP-3 is an oxytocin/OT/AVP antagonist, and has some selectivity in receptors of liver cells (Pettibone et al., 1989). It seems that its enhancement to HCV IRES-dependent translation would be more seriously in HCV patients, which should give more attention in clinic, as well as its analogs. However, in respect to drugs contraindications for HCV infected patients, few available reference could be found. Thus, combining the present and other screening systems, we could find clinical drugs that might stimulate HCV IRES-dependent translation to guide the clinical medication.

In summary, the HTS system established in this study could be effectively performed to find potential inhibitors and enhancers of HCV, which is helpful for the researchers to understand the replication of HCV and screen novel antiviral drugs in the future.

## Author Contributions

YZ and CS conceived and designed the experiments. YZ performed most of the experiments. PH, XL, and HD purified the natural products used in this study. NY and RL helped to do the high throughput screening experiments. YZ and CS analyzed the data. YZ and CS prepared the figures and wrote the paper. LZ and FS helped to write the manuscript. All authors reviewed the manuscript.

## Conflict of Interest Statement

The authors declare that the research was conducted in the absence of any commercial or financial relationships that could be construed as a potential conflict of interest.
